# Proposal: Bold New Indications for Transcatheter Pulmonary Flow Restrictors

**DOI:** 10.1007/s00246-024-03759-4

**Published:** 2025-01-05

**Authors:** Dietmar Schranz

**Affiliations:** Pediatric Heart Center, Johann-Wolfgang-Goethe University Clinic, Theodor-Storm-Kai 7, 60596 Frankfurt, Germany

**Keywords:** MVP, Pulmonary flow restrictor, PFR, Hypoplastic left heart syndrome, HLHS, Borderline LV, Dilated cardiomyopathy, DCM, Treatment

## Abstract

This proposal presents a proof of concept for the use of pulmonary flow restrictors (PFRs) based on MVP™-devices, drawing from clinical experience, and explores their potential role in the management of newborns with hypoplastic left heart syndrome (HLHS), other complex left heart lesions, and infants with end-stage dilated cardiomyopathy (DCM). At this early stage of age, manually adjusted PFRs can be tailored to patient’s size and hemodynamic needs. Although currently used off-label, PFRs have substantial potential to improve outcomes in these vulnerable patient populations. When integrated into a holistic treatment strategy, they represent a promising advancement in care. There is significant potential to reduce mortality and improve the quality of life for neonates with HLHS and variants, as well as to support age-dependent functional regeneration in DCM, all while avoiding the need for advanced surgical procedures, including general anesthesia. The main barrier to broader adoption is the limited availability of appropriately sized MVP devices. However, with ongoing patient-centered knowledge sharing and refinement of transcatheter techniques, there is reason for optimism that PFRs, tailored for individual patients, could provide significant benefits to thousands of infants with congenital heart disease and DCM worldwide.

**Supplementary Information:**

The online version contains supplementary material available at 10.1007/s00246-024-03759-4.

In 1952, Müller and Damman recognized the importance of creating artificial pulmonary artery stenosis, pioneering the surgical technique of pulmonary arterial banding (PAB) in a 4-month-old infant with a single ventricle physiology [1]. Surgical PABs, whether applied centrally or as bilateral pulmonary branch bands (bPAB), remains a key intervention in the palliation of congenital heart defects, particularly those associated with pulmonary overcirculation [2]. Since then, PAB has been incorporated into the Hybrid approach [3, 4] and is increasingly utilized as a novel technique to enhance ventriculo-ventricular interaction (VVI) in patients of heart failure and reduced left ventricular ejection fraction (HFrEF), particularly in infants with dilated cardiomyopathy, DCM [5]. The successful application of the Hybrid procedure in newborns with hypoplastic structures of the left heart [6, 7], along with the use of surgical PAB to address left ventricular dilation and dysfunction [5], has led to a paradigm shift in therapy. This shift emphasizes minimally invasive procedures for neonates with hypoplastic left heart syndrome (HLHS) and promotes regenerative strategies over organ transplantation, especially in infants with DCM. The combination of PA-banding and cardiac protective drug therapy has shown notable success leveraging age-and disease-related potential for cardiac recovery and functional regeneration [8, 9].

The Hybrid stage1procedure (S1P), as performed in Giessen, was designed to combine an open-chest surgical bPAB with percutaneous stent placement within the arterial duct. Unlike surgically performed PABs, which result in a circumferentially narrowing of the vessel lumen, percutaneous PAB (ePAB) was developed to reduce invasiveness. In vivo measurements indicate that narrowing the pulmonary trunk by approximately 50–60% compared to baseline [8] effectively increases flow velocity and generates pressure gradient across the PAB, without reducing resting blood volume, provided the subpulmonary ventricular remains functional and pulmonary artery compliance is preserved.

Bilateral PAB, as part of the Hybrid S1P in neonates with HLHS, was initially monitored using invasive pressure measurements across the banded pulmonary branch arteries.

The procedure was facilitated by the Marc Galantowicz method [7] which involved cutting PTFE-tubes with diameters of 3 or 3.5 mm, depending on infant’s weight (less than 3 kg or greater than 3 kg, respectively). From the outset, however, the goal was to create a percutaneously implantable endo-vascular pulmonary artery band (ePAB), suitable for use in neonates.

Early ePABs were crafted using Jo-med-graftmaster™ stents manually mounted onto hourglass balloons. The feasibility of implanting these devices—either as hourglass or dog-bone graft stents—was successfully demonstrated in a newborn sheep model via jugular vein access**.** However, clinical application faced challenges due to excessively large introducer sheaths, particularly for newborns with HLHS [10]. The first and second generations of the “Amplatzer™ flow limiter”, designed by Kurt Amplatz in 2000, were also successfully used in similar neonatal animal studies, including cases involving ductal stenting [10]. Despite these advancements, the ultimate goal remained to achieve a semi-invasive transcatheter S1P for neonates with HLHS.

Marc Boucek et al. used Amplatzer™ flow limiters combined with ductal stenting in patients with HLHS beyond the neonatal period, demonstrating their effectiveness as a bridge to heart transplantation (HTX) [11, 12].

The primary objectives of an endoluminal PFR are to reduce or maintain pulmonary blood pressure downstream of the device and to increase the pressure in the subpulmonary ventricle, either independently or in conjunction with reduced blood volume. Essentially, a PFR serves as both a flow limiter and a mechanism to sustain blood volume despite changing pressure conditions. For bilateral PFRs, it is crucial to assess the presence and function of a patent arterial duct (PDA), as this may play a role in regulating systemic blood flow. In some cases, the PDA may exclusively manage systemic blood flow, as seen in newborns with HLH-S, or only partially contribute to systemic blood flow, as seen in neonates with hypoplastic left heart complex (HLHC) or borderline left ventricular (BLV) morphology.

Therefore, PFRs should be specifically designed to regulate pulmonary blood flow without compromising the developing pulmonary vasculature. Additionally, they must also offer an alternative to surgical PAB to retrain either the subpulmonary right or left ventricle and enhance ventriculo-ventricular interaction (VVI).

Currently, percutaneous transcatheter PAB devices are under development as potential alternatives to surgical PABs. Animal studies have validated the efficacy of transcatheter devices for central PAB [13], and the feasibility of various transcatheter devices for bilateral PAB has been explored in both animal studies [10, 14] and clinical applications. These studies have shown promise in newborns with duct-dependent systemic blood flow [15], and even infants [16] and adults [17] with reduced left ventricular ejection fraction (HFrEF).

## MVP-Based PFR, Materials and Procedural Considerations

A groundbreaking advancement in the field of medical devices was marked by the introduction of the Medtronic® Vascular Plug™ (MVP, Medtronic Inc., USA), initially designed for neurointerventional procedures and other interventional radiology interventions [18]. This device has received FDA approval and carries the CE mark. Although not officially approved for the here described indication, there has been a growing trend toward using the MVP device to occlude arterial ducts safely and efficiently, particularly in premature infants [19].

The off-label use of MVP-based PFRs for bilateral endoluminal PAB was first reported in an animal study [14], followed by successful human use in newborns with HLHS and variants [15]. Since then, the potential of MVP-based PFRs has been demonstrated in multiple institutions worldwide [15, 20–26]. However, current published data reveal slight variations in handling techniques, procedural objectives, and learning curves, which may reflect differences in institutional approaches to managing neonates with hypoplastic left heart structures that rely on duct-dependent systemic blood flow, or infants with significant heart failure resulting from left–right shunts [23] or left ventricular dysfunction [16]. Despite these variations, the emerging insights into the challenges and benefits of MVP-based PFRs highlight their promising role as part of a comprehensive treatment strategy.

The MVP’s unique design, originally intended to seal vessels in a low-dynamic, “low-pressure” environment, enables insertion through 4Fr and 5Fr diagnostic catheters. This makes the device particularly suitable for the effective application of a manually prepared PFR in neonates with HLHS and related variants, representing a significant advancement in clinical practice.

The MVP features a self-expanding nitinol cage, partially covered proximally by an ultra-thin PTFE (polytetrafluoroethylene) material, while the distal taper remains uncovered. Two radiopaque platinum markers positioned at each end of the cone-shaped device enhance its visibility during fluoroscopy. The device is mechanically attached to a 180 or 165 cm-long flexible delivery cable via screw mechanism.

Currently, MVPs are available in four model specifications:

- MVP-5Q: This model has eight covered end segments and an unconstrained outer diameter (OD) of 6.5 mm. It is recommended for occluding vessels ranging from 3.0 to 5.0 mm in diameter. When constrained within a microcatheter with a minimum diameter of 0.027 inches, the MVP-5Q has a maximum length of 16 mm and an unconstrained length of 12 mm.
- MVP-7Q and MVP-9Q: These models each consist of 10 covered segments. The MVP-7Q has an unconstrained length of 16 mm and expands to a diameter of 9.2 mm, making it suitable for occluding vessels from 5.0 to 7.0 mm in diameter. The MVP-9Q has an unconstrained length of 18 mm and expands to a diameter of 13 mm, ideal for vessels with diameters ranging from 7.0 to 9.0 mm.

Given the high-flow and high-pressure characteristics of the pulmonary arteries, it is critical that MVP-based PFRs are oversized relative to the measured vessel lumen to prevent distal migration. However, excessive oversizing should be avoided, as this can lead to material deformation or compression of surrounding structures, such as the small ascending aorta in HLHS [23, 25, 26].

Current clinical experience does not yet provide definitive, universally applicable guidelines for the optimal manipulation of MVPs for efficient PFR implantation. These limitations are likely due to the relatively limited experience with MVP-based PFRs and the variations in age- and disease-dependent indications and therapeutic goals.

**Fig. 1:**
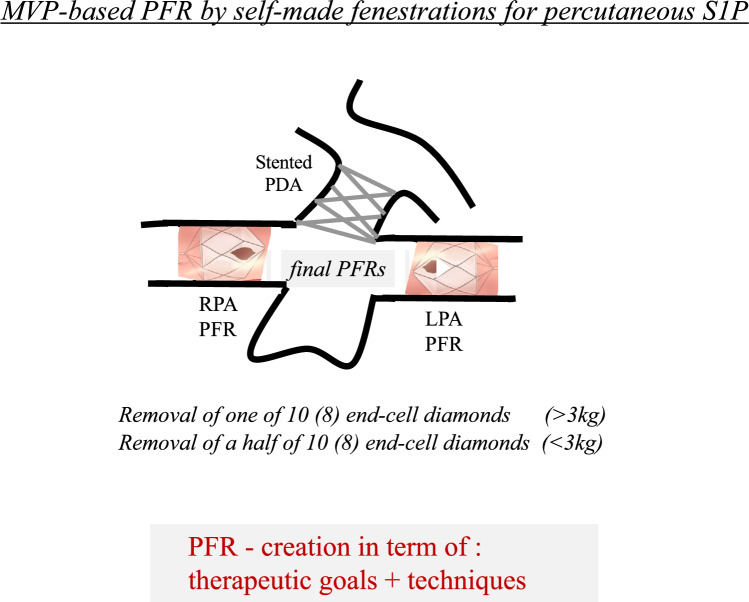
Illustration of MVP-based pulmonary flow restrictors (PFRs) with differently fenestrated, PTFE-covered end-cell resections positioned in the right (RPA) and left (LPA) pulmonary branch arteries. A schematic of a ductal stent is also shown

To address these challenges, the technique used in Frankfurt [26] is described, which has been slightly modified and further refined for individualized applications of MVP-based PFRs in neonates with HLHS/HLHC (Fig. 1) and in young infants with DCM.

This innovative PFR-based approach integrates key insights from the Giessen Hybrid approach [27] and incorporates lessons learned from the use of PAB in patients with left ventricular dilated cardiomyopathy (LV-DCM), particularly those with preserved right ventricular ejection fraction in young infants [28]. Both applications are supported by a comprehensive peri-interventional treatment strategy designed to optimize outcomes.

Table 1 summarizes this holistic approach [29], which includes considerations for adjunctive drug therapy, representing a transformative treatment paradigm for newborns with HLHS.

**Table 1:** Holistic Percutaneous Stage-1-Procedure (S1P) in Neonates with Hypoplastic Left Heart Syndrome (HLHS)

Step	Intervention/Description	Comments/Additional Notes
1. Prenatal Diagnosis and Intervention	Perform precise prenatal diagnosis, with interventions as necessary and available	Early identification improves management planning
2. Maternal Delivery Planning	Carefully plan and organize the maternal delivery	Optimizes delivery for neonatal well-being
3. Postnatal Mother-Infant Bonding and Monitoring	Facilitate mother-infant bonding; monitor heart rate, pulse oximetry, intermittently measure systolic and diastolic blood pressure	Essential for early detection of anomalies
4. PGE1 Infusion	Place a peripheral intravenous line for continuous low-dose PGE1 infusion (5 ng/kg/min)	Critical for maintaining ductal patency
5. Echocardiography	Perform echocardiography for accurate morphological and hemodynamic assessment	Required for comprehensive assessment of cardiac function
6. Oral Feeding	Allow ad libitum oral feeding	Promotes normal growth and development
7. Bisoprolol (ß1-Blocker)	Administer 0.1 mg/kg bisoprolol oral, once daily	Supports hemodynamic stability
8. Parental Consent	Obtain written parental consent for percutaneous S1P, including off-label use of MVP-based pulmonary flow restorers (PFRs)	Essential for legal and ethical considerations
9. Percutaneous S1P (Transcatheter Procedure)	Perform bilateral PFR placement followed by ductal stenting on day 2 or 3. The procedure should be done when the infant is stable, non-hungry, and mildly sedated	Timing and sedation status are critical for safety
10. Post-Procedure Transfer	Transfer the infant back to the mother–child unit with intermediate care facilities for further monitoring	Ensures appropriate postoperative care and observation
11. Continued PGE1 Infusion and Heparin	Continue low-dose PGE1 infusion, combined with an intravenous heparin infusion (300 IU/kg/day)	Prevents thrombosis while maintaining ductal & isthmus patency despite duct stent
12. Medication Transition (day 2–3 post-S1P)	Discontinue PGE1 and heparin; begin ASA (1–2 mg/kg) and clopidogrel (0.2 mg/kg), with continued oral bisoprolol	Transition to interstage medications based on clinical and echocardiographic weekly re-evaluation

## Transcatheter Procedure, Preparation of the PFR and Deployment

Elective percutaneous PFR implantation is usually performed on spontaneously breathing patients under light sedation, regardless of age or underlying disease [26–28]. A well-prepared team, equipped with standard non-invasive monitoring and an adequate supply of materials, is essential to the success of the intervention. Antibiotic prophylaxis is administered prior to the procedure while the patient is still on the ward. Anesthesia is generally avoided, especially in elective treatment of newborns with duct-dependent systemic blood flow who are not intubated due to the illness [23, 24].

Following femoral vessel puncture and sheath insertion, systemic heparinization is administered as a single intravenous dose of 100 IU/kg. A second dose of 50 IU/kg of heparin is given after PFR placement, without clotting time monitoring (ACT). Typically, a 4-Fr Terumo® sheath is placed in the femoral artery and a 5Fr Terumo sheath into the vein. A 6Fr venous sheath is reserved for additional interventions, such as Rashkind balloon atrial septostomy. Biplane fluoroscopy is strongly preferred for percutaneous transcatheter S1P. PFR placement is generally recommended before ductal stenting, provided that duct obstruction is ruled out despite PGE1-treatment.

A straightforward and brief transcatheter procedure is advisable, not only for neonates with HLHS but also for young infants with DCM, where the goal is to improve ventriculo-ventricular interaction (VVI). Unnecessary invasive diagnostics procedures before or after the procedure should be avoided.

For PFR implantation, a 4-Fr non-tapered or Cobra-shaped 4 or 5Fr Radiofocus® glide catheter (Terumo® Corp, Japan) with a minimum lumen diameter of 0.0038″ is primarily used. A 4Fr Judkins catheter (0.0035″) is suitable for placing an MVP-5Q-based PFR. Right (RPA) and left PA (LPA)-branches are visualized through selective angiographies in 30° right anterior oblique (RAO) and 30° left anterior oblique (LAO) projections, respectively [26].

**Fig. 2:**
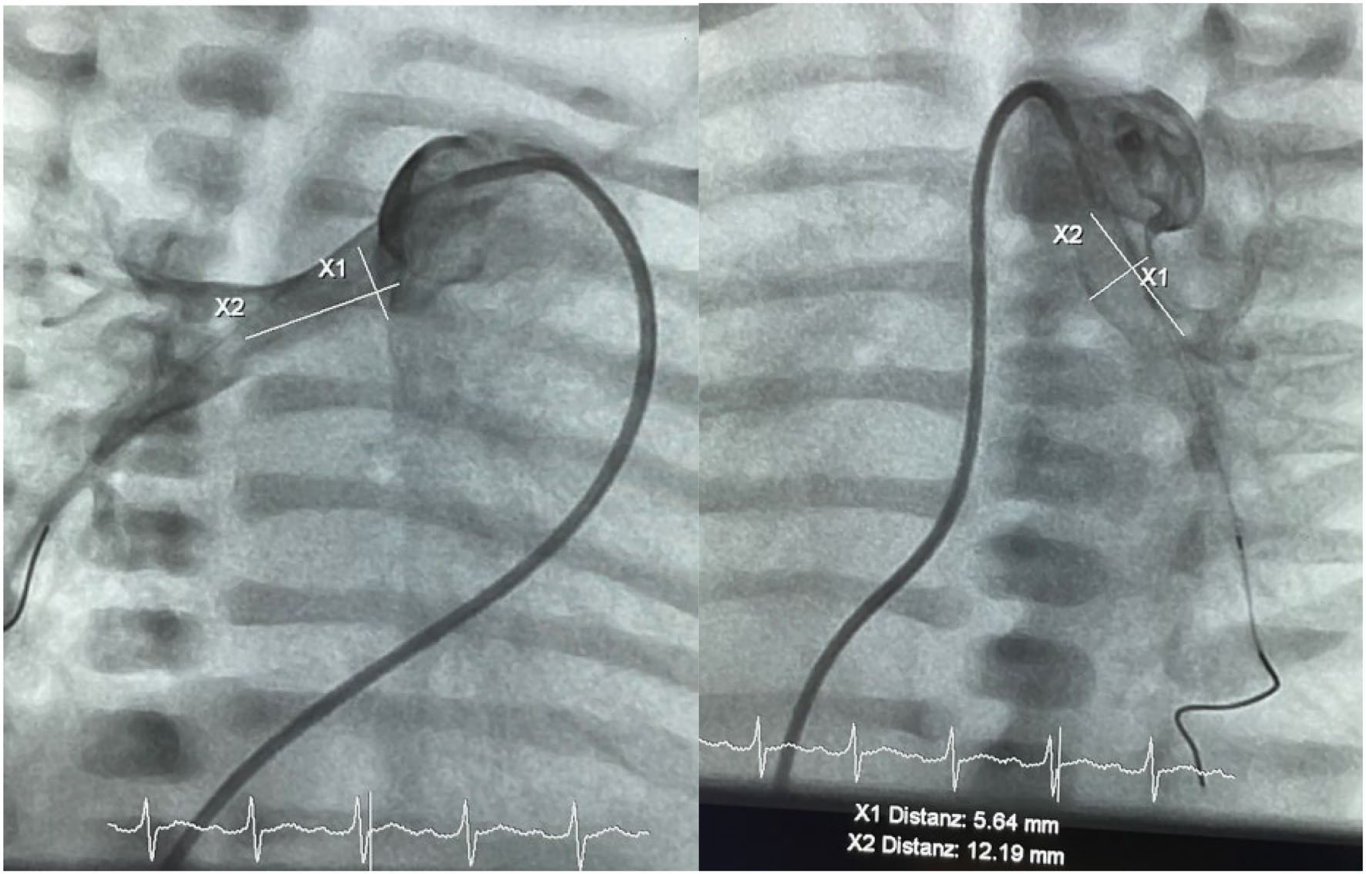
**a** Angiographic image of the right pulmonary artery (RPA) in right anterior oblique (RAO) 30° projection, showing the measured width and approximate length of the RPA from the entry point to the distal bifurcation. **b** Angiographic image of the steeply angled LPA in left anterior oblique 30° (LAO) projection, used for measuring the vessel dimentions

The precise anatomy of the PA-branch is exposed through small hand injections of contrast medium via an attached Y-connector, while the catheter is retracted over the distally fixed guidewire (Fig. 2a, b).

**Fig. 3:**
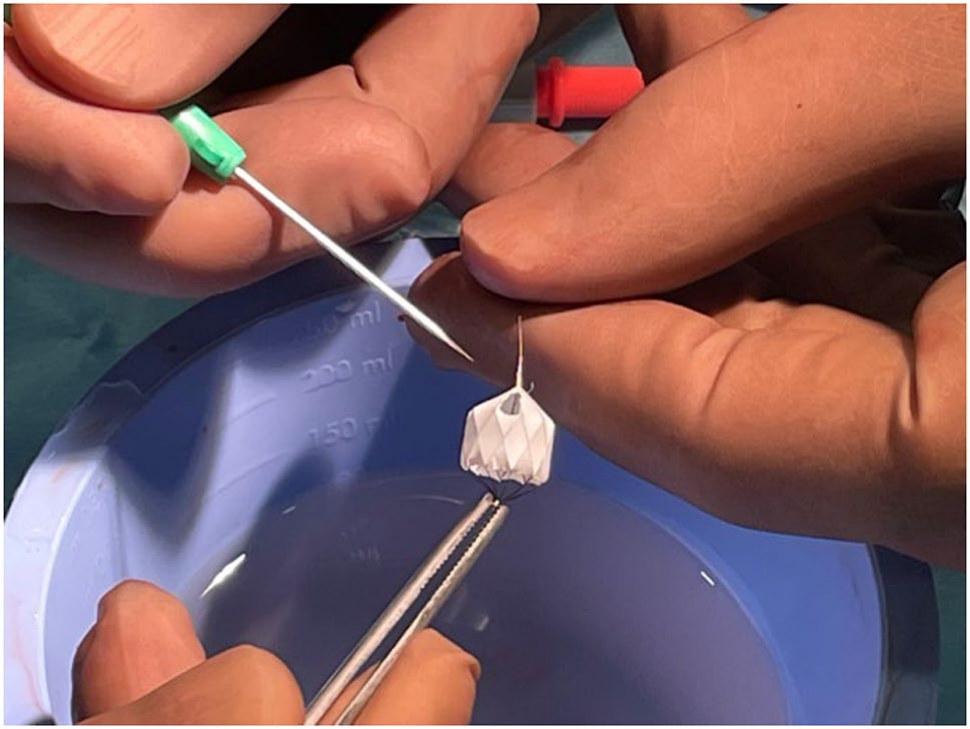
MVP-7Q device held with tweezers after the creation of a defined fenestration using an eye scalpel. Approximately half of one of the ten PTFE-covered end-cells in the tapered part of the device is resected

The angiographically determined dimensions of the pulmonary artery branches—specifically the diameters and lengths form their origin to the peripheral bifurcation—guide the selection of an appropriate MVP size. Once, the correct MVP size is determined, the catheter is advanced distally to ensure stable positioning while the MVP is manually prepared into a PFR. One operator secures the distal radiopaque marker with anatomical forceps, while another operator holds the delivery cable at the proximal covered end of the MVP. The PTFE membrane within the selected diamond end cell is cut, preferably using a fine cornea scalpel (Fig. 3).

The extent of PTFE resection depends on the age-related diameters of the pulmonary artery branches as well as the specific indication and therapeutic goal for PFR use. Typically, this involves removing all or half of the PTFE-covered end-cell, but not extending all the way to the Nitinol-frame, as illustrated in Figs. 1 and 3.

**Fig. 4:**
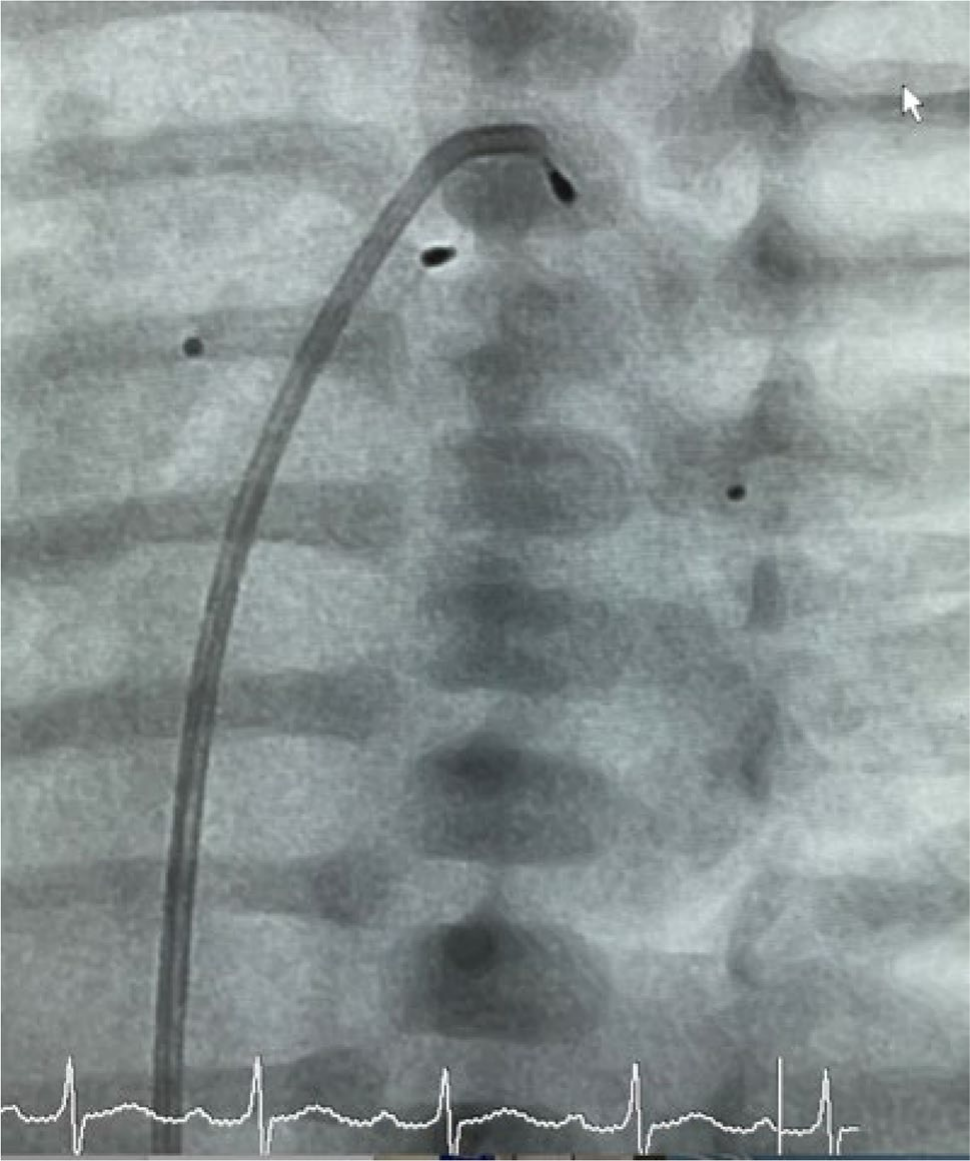
Optimal positioning of the PFR at the entry of the LPA, still attached to the delivery cable

After the manual conversion of the MVP to a PFR, the device is immersed in heparinized 0.9% saline and then withdrawn into a loading catheter. It is subsequently inserted through the flushed Y-port connected to the glide catheter. Re-sheathing of the device—whether in vitro as in vivo—after manipulation into a PFR, can potentially damage to the PTFE material. Excessive contact between the MVP-based PFR and the delivery catheter may cause a “cobra-phenomenon” of the Nitinol-based cage, inhibiting the self-expanding mechanism. The wireless glide catheter remains positioned distally in the PA branch. Once the distal radiopaque marker aligns with the tip of the guide-catheter, the catheter containing the PFR device is cautiously withdrawn and partially expanded. It is then fully expanded to the marked branch bifurcation point. The marker is held in place by the introducer cable, and the catheter is removed while the nitinol-PFR expands under fluoroscopic guidance **(**Fig. 4). The uncovered distal portion of the MVP enhances the device’s, performance by minimizing the risk of obstructing the upper lobe branches (see, Supplementary Video 1, 2, and 3).

**Fig. 5:**
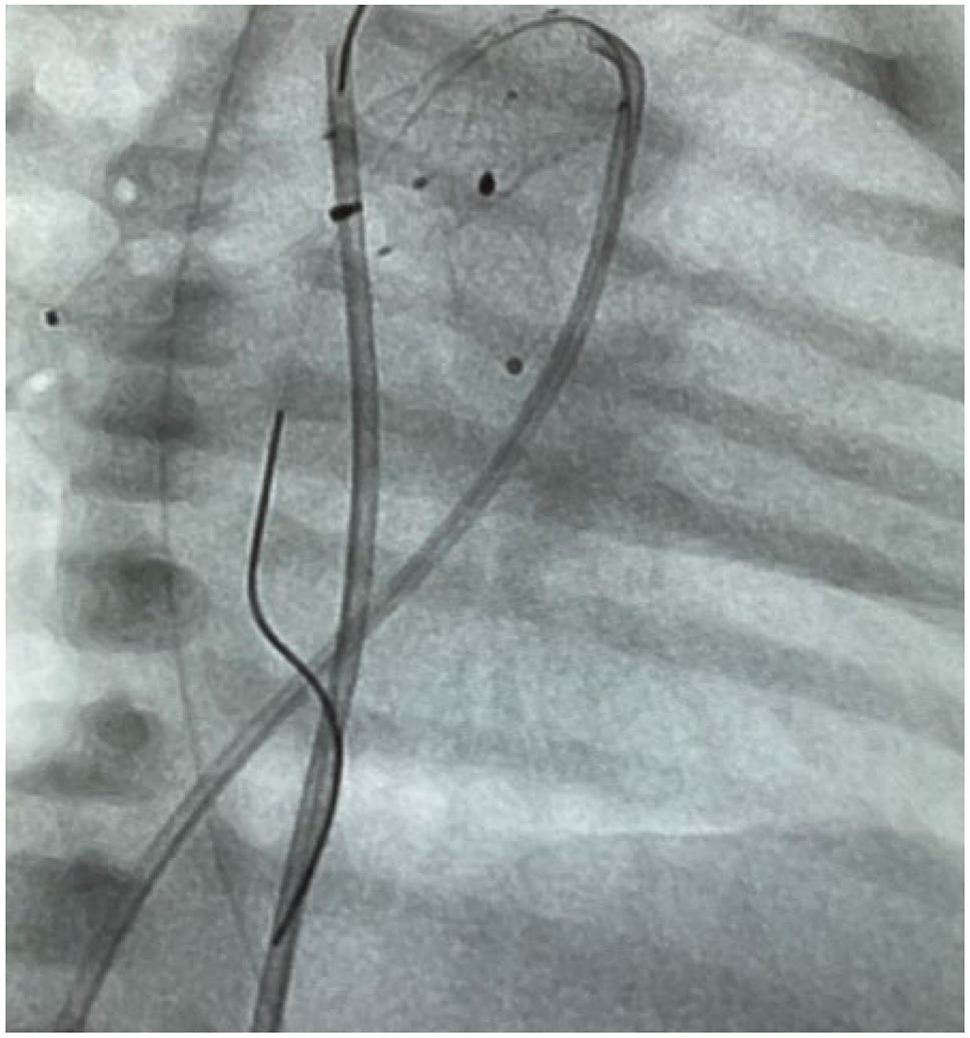
Completed percutaneous stage-1-procedure (S1P) with bilateral PFRs and a ductal stent, captured in right anterior oblique (RAO) 30° angulation. The final evaluation shows the junction of the duct and descending aorta using a multipurpose 4Fr catheter and floppy guidewire, which remains in the descending aortic arch for pressure measurements and manually contrast medium injection. Additionally, a 4Fr Judkins catheter, guided by a coronary wire, is positioned in the descending aorta via stented duct

To prevent proximal migration of the PFR-device toward the pulmonary trunk and to confirm its correct positioning, it is essential to ensure the device is fully expanded. Final positioning is confirmed by injecting contrast medium through the delivery catheter to assess adequate blood flow into the upper and lower lobe arteries before releasing the device. Once PFRs are placed in both pulmonary artery branches, the percutaneous S1P is completed with the placement of ductal stents, followed by a final assessment of the duct-descending aortic junction (Fig. 5, Supplementary Video 4).

**Fig. 6:**
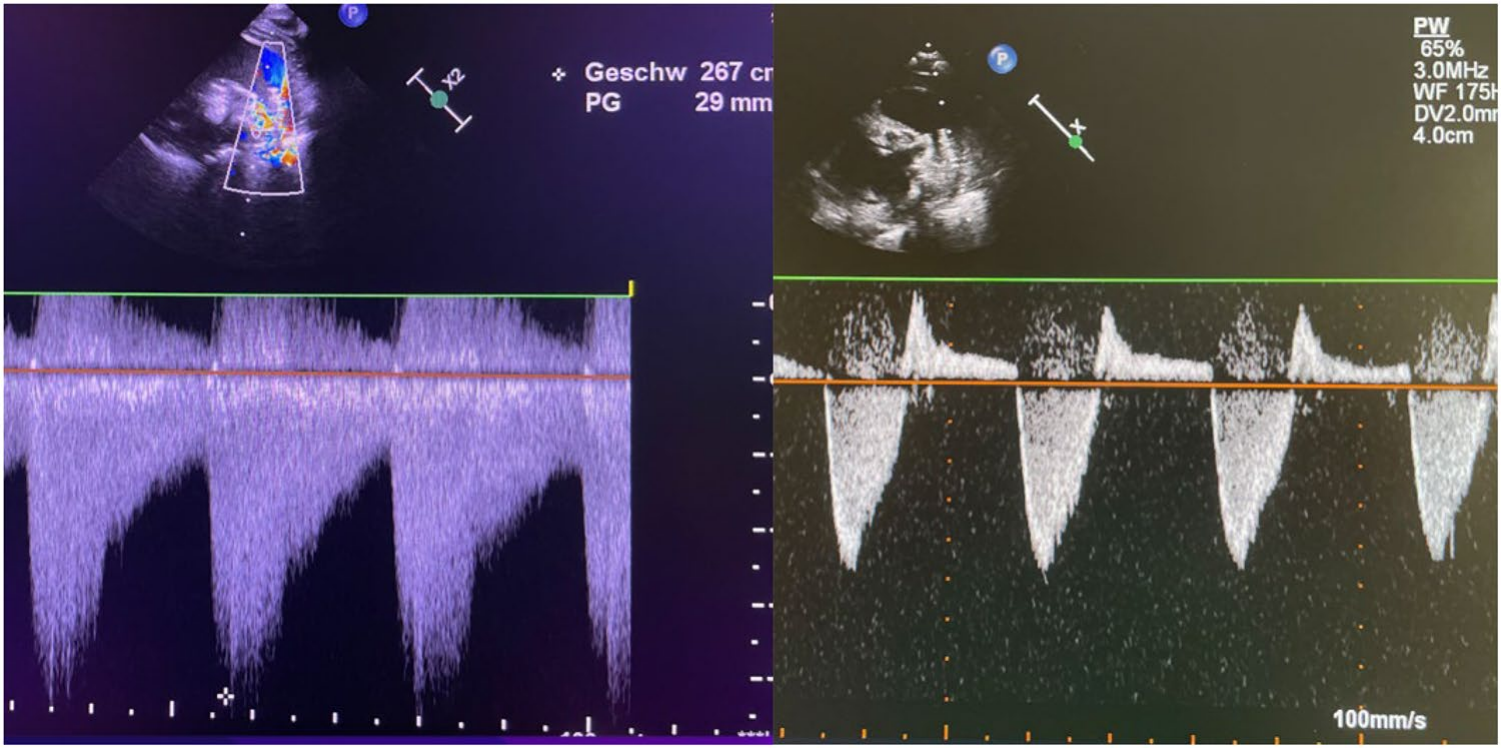
**a** Final evaluation after percutaneous stage 1-procedure in a newborn with hypoplastic legt heart syndrome (HLHS). Color and CW-Doppler signals are obtained over a pulmonary flow restrictor (PFR) positioned in the right pulmonary artery (RPA). The systolic-diastolic Doppler profil indicates effective PFR function. Low perpherial pulmonary pressure is suggested by the maximum systolic velocity, while the approximately one-third ratio of the diastolic to systolic flow velocity corresponds to obstruction and effectiveness of the PFR. **b** Doppler flow analysis through a stented duct (8 × 20 mm Sinus-Superflex-DS). The systolic flow pattern is laminar indicating unobstructed blood flow. The slightly increased Doppler velocity is typical of a stented vessel, which is non-compliant. The corresponding diastolic left-to-right shunt is well tolerated and results from adequate bilateral pulmonary branch banding, effective right ventricular function, and pulmonary valve competency. Additionally, ß1-receptor blocker therapy maintains an appropriate heart rate, as evidenced by a normal systolic-to-diastolic time interval in the Doppler flow profile. These combined factors ensure sufficient systemic blood flow, assuming the retrogradely perfused aortic arch and the coronary and cerebral arteries remain unobstructed

Before removing the femoral access sheaths, a final transthoracic echocardiographic examination is conducted to assess heart function, heart valves, and the position and effectiveness of the PFRs, using color and continuous wave (CW) Doppler. In newborns with HLHS, the adequacy of the implanted PFRs can be identified and assessed using a systolic-diastolic Doppler profile (Fig. 6a). The ratio of the systolic-to-diastolic portion of the Doppler curve reflects the magnitude of the obstruction.

However, in the assessment of an efficient transcatheter S1P is more than just demonstrating a systolic-diastolic flow profile across the PFRs. It also includes evaluating the ratio of systolic right-to-left versus diastolic left-to-right Doppler flow patterns through the stented duct (Fig. 6b), as well as the retrograde flow characteristics of the aortic arch in the context of the univentricular cardiac function.

## Post-procedural Care and Follow-Up

After the percutaneous transcatheter procedure, electively treated patients are transferred back to the intermediate care ward, where a mother–child unit facilitates reunion of the mother with her child [29]. According to our protocol, treatment typically involves continuing an infusion of heparin (300/IU/kg/day) for at least two days. In newborns with duct-dependent systemic blood flow and a stented arterial duct, heparin is combined with the uninterrupted continuous infusion of PGE1 at a dosage of 5 ng/kg/min for an additional 1 to 2 days. If the child exhibits good oral feeding behavior, a single dose of oral clopidogrel (0.2 mg/kg/day in neonates) and low dose acetylsalicylic acid (ASA, 1-(2) mg/kg/day) is administered. This aims to preferentially block the platelet thromboxane production over endothelial prostacyclin, with hypothesizes of avoiding arterial duct constriction while potentially reducing in-stent proliferation.

Following discharge, routine follow-up visits are scheduled every eight to ten days especially after transcatheter S1P. If necessary, MVP-based PFRs can be easily removed and re-implanted within the first few days and weeks post-implantation, provided appropriate capture loops and 4 or 5F long sheaths are used. However, it is important to note that harvesting a PFR with an open right-to-left shunting duct carries a potential risk of systemic embolization of thromboembolic and proliferative material.

Typically, PFRs are surgically removed during comprehensive stage 2 palliation (S2P) or when biventricular repair is postponed due to this novel percutaneous S1P technique.

## PFR-Technique and Follow-Up in Neonates and Infants with DCM

Given the current limited size of MVP-based PFR, their use it’s still restricted to neonates and young infants with left ventricular DCM with preserved right ventricular function. The transcatheter technique is much less traumatic for these children and requires significantly less perioperative and intensive care effort. As with the transcatheter S1P technique in neonates with HLHS, non-intubated infants with DCM are typically treated using light sedation and local anesthesia. The success of bilateral endoluminal PAP in the vulnerable end-stage DCM patient cohort depends also on a straightforward approach that addresses on addressing the most urgent needs. Monoplane fluoroscopy is sufficient for placement of bilateral PFRs. The decision to place the first PFR in the RPA or LPA depends on several technical and anatomical factors. Notably, the diameter of the contralateral PA-branch generally increases after successful placement of a PFR in the first branch. Given the typically borderline wide diameter (up to 9 mm) using the currently largest MVP-9Q based PFR device in an infant weighing around 6–7 kg, the RPA is usually selected first, [16]. An effective PFR can typically be achieved by cutting half of one of a MVP-9Q PTFE-covered end-cells. It is important to recognize that growth into the PABs can still occur in infants with severe DCM, and overly tight bands may not be immediately necessary unless the procedure is urgent.

To prevent inadvertent device migration, the conical bare metal portion of the device can be positioned at the entrance of the upper lobe branch, as described in neonates with HLHS and variants [25]. Due to the current lack of larger MVP-based devices, this technique might particularly be useful in older infants with DCM for whom PA banding is considered, but surgery is not an option. While placement of two devices—one in the upper and one in the lower pulmonary artery peripheral branches—has been considered, it has not yet been performed for effective endoluminal PAB in infants with DCM.

After successful placement of the endoPAB, the antiaggregating treatment is similar to that of newborns following S1P. However, the clopidogrel dosage is age-dependent slightly higher than 0.2 mg/kg/day indicated in newborns.

In our initial series of three infants with left ventricular DCM, functional cardiac regeneration developed in all patients following transcatheter bilateral PABs [16]. Partial transcatheter de-banding was successfully performed at ages of approximately 3 years, 15 months, and 12 months, respectively. Gradual balloon dilations were performed by coronary balloons (4 × 15 mm) followed by larger balloons such as the Tyshak-Mini™ (6 × 20 mm) or high-pressure balloons. Considering that all currently by transcatheter technique treated patients, suffered non-compaction-associated DCM only partial percutaneous de-bandings were performed aiming an elevated right ventricular pressure that remained around 30–50% of the systemic level even in the long term [28].

**Fig. 7:**
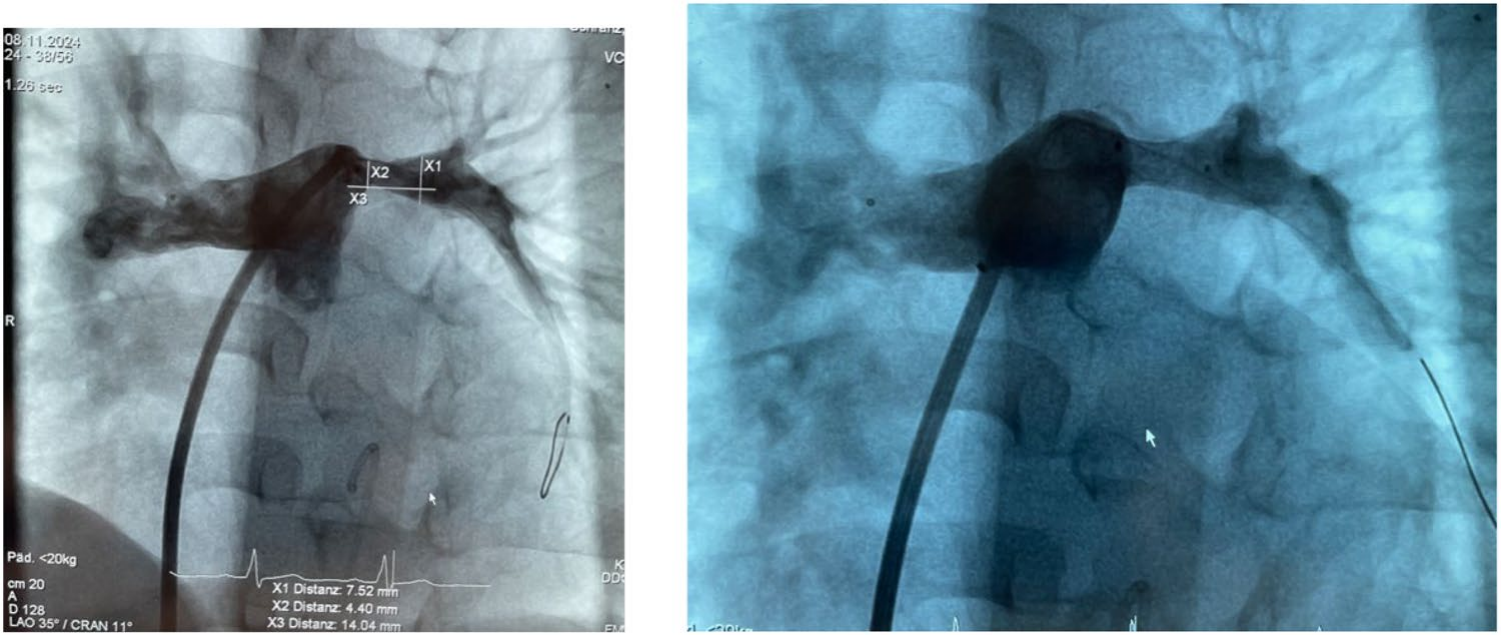
**a** Main pulmonary artery angiography via manual contrast medium injection through a 5Fr Flexor-sheath®. In the dilated right pulmonary artery, the distal marker of the MVP-9Q based PFRs is visualized in the entrance of the right upper lobe, while the left pulmonary artery shows a significant obstruction. Based on the measurements and gradual pre-dilations by coronary and Tyshak-Mini balloons, a Formula 6 × 112 mm was ultimately implanted (**b**). The residual systolic pressure ratio between the right and left ventricle was 39 mmHg to 97 mmHg, respectively

Figure 7a, b show MVP-9Q based PFRs after a follow-up period of 5.5 years in the first, now exactly 6 years old former infant with end-stage non-compaction DCM.

The PFR shown in the RPA was replaced when the first implanted device was removed by transcatheter snare technique due to insufficient efficacy, four months after the initial endoluminal banding procedure. The second MVP-9Q with a resected half-covered diamond end-cell, was implanted with anti-migration technology, positioning the tapered uncovered distal MVP part at the entrance of the upper lobe. During follow-up, as the left ventricle function regenerated and the NYHA functional class (FC) IV normalized to FC I, both PFRs were gradually re-dilated twice to avoid complete pulmonary de-banding due to the non-compaction structure [16]. However, over time a pulmonary perfusion mismatch in favor of the RPA developed, leading to a stent implantation within the MVP-based PFR (Fig. 7b). Independent of this anecdotal report of a percutaneous removal and reimplantation of a MVP-based PFR four months after implantation, the current experiences in animal studies [14] and human use [15, 23–26] definitive removal of manually created PFRs by transcatheter snare techniques is not predictable after an implantation period of almost two months. However, surgical removal or placement of a stent provided as sufficient strategies to eliminate acquired PA-branch obstruction, if necessary. However, in all three patients, non-compaction associated DCM indicated an increased right ventricular pressure of about 30–50% of the systemic level even in long-term [28].

## Current Results Using Pulmonary Flow Restrictors

Published experiences with MVP-based PFRs endovascular pulmonary branch banding are still limited. However, no procedure-related mortalities have been reported to date. Several pediatric heart centers have adopted this novel approach for selected high-risk patients, including newborns with HLHS weighing less than 2.5 kg. Transcatheter endoluminal PA banding procedures could replace or delay advanced neonatal surgeries or even avoid open-chest approaches, which may be reduce both the risk and total number of surgeries [15, 20–26]. Term, and preterm newborns with HLHS and HLHC, young infants with syndromes associated congenital heart defects including left-to-right shunt-related pulmonary overcirculation, have been successfully palliated using this novel method [20–26]. Personal experiences presented at the AATS, in Toronto 2024 (see open access video clip) across various pediatric cardiac institutions in Germany indicate successful percutaneous S1P in 27 newborns with HLHS and variants with no reported mortality. However, three patients died during the interstage period at home, two of them during the COVID-19 pandemic. The percutaneous transcatheter approach significantly improved the quality of life for all treated newborns by reducing postnatal hospital stay and enabling almost normal mother–child bonding during the postnatal period and during the follow-up at home, as proposed [29].

Additionally, percutaneous transcatheter S1P has been successfully used as a bridge to definitive surgery in neonates with severe congenital aortic valve stenosis complicated by left ventricular heart failure and severe mitral valve regurgitation.

To date, nine young infants with DCM and severe heart failure have been palliated with bilateral pulmonary artery endo-banding, three of whom were treated outside of Germany. While all patients showed clinical improvement, the first two remained listed for heart transplantation and unfortunately died during follow-up, one after undergoing surgical mitral valve repair. AS mentioned above, five patients with appropriately sized pulmonary branch arteries fully recovered with the currently available MVP-based PFR sizes.

Three patients abroad with pulmonary arteries already too large for an immediate, optimal PFR effect, were treated due to the lack of alternative options. All three showed clear clinical improvement, though complete functional regeneration has yet to occur in one of them.

Medtronic microvascular plugs modified as PFR have also been employed to alter both MAPCA and duct-dependent pulmonary blood flow [30], or to manage stent flow in an overly fenestrated Fontan circulation originating from an extra-cardiac conduit [31].

## Final Remarks and Future Directions

The flexibility of manually performed MVP-based PFRs, which allow tailored fenestrations, makes them attractive for individualized applications. These devices hold significant potential for novel percutaneous transcatheter treatments of complex heart diseases.

Regardless of the technique used—scalpel, punch, laser, cautery, or other methods—effective endovascular PA-bands are crucial. Optimal PFR placement depends on adequate device selection, delivery process and implantation technique. It is desirable to confirm angiographically optimal PFR positioning, including the relationship between device and vessel dimensions, as well as the quality of distal pulmonary artery perfusion, especially the upper lobe branches, before releasing the device. This ensures tangential alignment of the PFRs during high blood flow in the pulmonary artery, reducing the risk of distal device migration. In newborns with an extremely small ascending aorta (AAO), placing a right-sided PFR distally—even with an unprotected right upper lobe—can be an accepted strategy to avoid potential AAO compression by an oversized PFR in the right pulmonary artery branch.

On the other hand, as noted earlier, inadvertent device migration can be managed by placing the tapered bare metal portion at the entry of the upper lobe branch [25]. Due to the current lack of larger MVP-based devices, we have used this technique in older infants with DCM (Fig. 7a). Furthermore, for older infants with DCM who are not candidates for surgical PAB, double device placement, in the upper and lower pulmonary arteries on one side may be considered.

The efficiency of bilateral PFRs is reflected in body weight gain, reduced cardio-vascular workload, particularly in neonates and infants with single ventricle pathophysiology. Pulmonary to systemic blood flow (Qp/Qs) should be balanced, the ratio of oxygen supply and consumption should be stabilized, and the growth of the pulmonary vascular bed should not be inhibited. Sufficient transpulmonary blood flow is essential for final Fontan circulation as well as in neonates with biventricular physiology and borderline left heart structures to achieve adequate oxygenation with adequate left ventricular preload for further ventricular growth. Thus, the placement of bilateral PFRs should be part of a holistic treatment plan that includes pathophysiological considerations as well as maternal bonding, stress reduction (e.g. oral feeding), and prophylactic treatment with ß1-specific beta-blockers [29].

The extent of vascular obstruction achieved by PFRs after percutaneous S1P is demonstrable by the systolic-diastolic Doppler flow profile, regardless of whether the fenestrations was achieved by resection of an entire or half of the PTFE-covered end-cell diamonds of the MVP device. This may also be due to an asymmetrical positioning at the proximal conical end and the partial lack of contact with the vessel wall. The diameter of the non-contacted fenestration is likely to be minimally affected unless the device is excessively oversized. Several factors influence the requirements for fenestration, including body weight, single or biventricular (patho-) physiology, and the presence, diameter, and function of a right-left shunting arterial duct. A well-planned periprocedural treatment strategy is essential.

For patient safety, the elective implantation of bilateral PFRs as part of percutaneous S1P under routine general anesthesia with intubation and ventilation should be reconsidered. General anesthesia-based transcatheter S1P might significantly impact vulnerable hemodynamics and ultimately PFR properties. Scenarios such as pulmonary overcirculation prior to PFR placement or seemingly reduced PFR efficiency afterward, could lead to minimized fenestrations, affecting pulmonary vascular development and causing disproportionate single ventricle hypertrophy. Additionally, the effects of excessive blood flow and pressure on the thin PTFE membrane need to be considered. Focusing on a MVP fenestration as small as possible is neither necessary nor desirable. When structural reasons or reasons or mechanical improvements are not indicated, appropriate drug therapy (e.g. prophylactic ß1-specific beta-blocker) can compensate for Qp/Qs imbalances and prevent myocardial dysfunction.

Two decades ago, bilateral surgical pulmonary PA-branch banding was performed using complicated pressure gradient measurements. The method by Galantowicz, using 3 or 3.5 mm PTFE-tubes, superseded these pressure measurements and simplified the entire surgical component of the Hybrid approach [7]. In the 1990s, institutions like Giessen listed newborns with HLHS primarily for HTX. These patients received ductal stents instead of continuous prostaglandin E1 infusion, but without bilateral PA-banding, while waiting for a donor heart, even at home. The main difference between the survivors who received transplants after waiting for 4 to 6 months and those who are now being bilaterally banded was the loss of body weight gain. Today, following percutaneous S1P, infants achieving age-appropriate weight can proceed comprehensive stage 2 surgery determined at an age of [3] to 4 months. Therefore, even an unprotected left or right upper pulmonary lobe branch—due to a too distally positioned or migrated PFR—does not preclude a successful three-stage procedure. Adequate pulmonary vascular development without right ventricular dysfunction even facilitates Fontan completion at ages two and three years. All HLHS patients who underwent complete percutaneous S1P [15] have since undergone an uneventful Fontan operation. It appears more advantageous to ensure the largest possible, but still effective, fenestration with a preserved systolic-diastolic Doppler profile.

In general, catheter procedures in patients with mixed shunt lesions, particularly those with single ventricle physiology and a right-to-left shunting duct, should be kept as short and simple as possible. Potential risks of right-left embolization of air, blood clots, or microparticles are associated with intravenous administration of medications, exchange of guidewires, or flushing of catheters.

Measures such as stent implantation in the arterial duct [32] or careful PFR positioning is adequate. Re-transversing MVP-based PTFE fenestration should be avoided to prevent inadvertent injury to the PTFE membrane. The thin and fragile membrane offers advantages when the PFR function is no longer required; restrictive PFR function can be even gradually relieved by balloon dilatation or stent placement, similar to transcatheter de-banding of surgically placed PABs.

From a user and applicability perspective, MVP-based PFRs have nearly ideal characteristics, although there is still room for improvement, particularly in device sizes for all age groups. The flow characteristics through MVP-based PFR are intriguing, with harmoniously contrasting angiographic images of wide-open pulmonary branches and minimal angiographic jet flow characteristics. Further investigation is needed to determine if these flow characteristics minimize thrombus formation compared to a device with centered jets, and whether the inside-out properties of the endoPAB reduce vascular damage to a single endothelial lesion, potentially making them superior to surgical bPAB used in the Hybrid approach. While current experience with the fate of the pulmonary branch arteries after removal of endo-banding by MVP-based PFRs is limited, the results are cautiously optimistic.

## Conclusion

The introduction of new technologies and clinical approaches requires careful consideration, particularly given the limited experience and the associated learning curve. However, the expanding multi-center experience suggests that transcatheter MVP-based PFR may signal the beginning of a new era in congenital and acquired heart disease treatment, with the potential for significant improvements in patient care, as supported by early hypotheses and comparisons with current procedural outcomes.

There is growing optimism that medical device companies will recognize the need to develop customized PFRs. Such innovations have the potential to address the unique needs of diverse patient groups, ranging from premature babies with univentricular heart diseases to older infants, adolescents, and even adults with life-threatening heart failure worldwide.

## Supplementary Information

Below is the link to the electronic supplementary material.

Supplementary file1 (MOV 11538 kb)

Supplementary file2 (MOV 4766 kb)

Supplementary file3 (MOV 4940 kb)

Supplementary file4 (MOV 5827 kb)

## Data Availability

No datasets were generated or analysed during the current study.
